# Decline in attrition rates in United States pediatric residency and fellowship programs, 2007–2020: a repeated cross-sectional study

**DOI:** 10.3352/jeehp.2025.22.24

**Published:** 2025-09-05

**Authors:** Emma Omoruyi, Greg Russell, Kimberly Montez

**Affiliations:** 1 Department of Pediatrics, McGovern Medical School at the University of Texas Health Science Center at Houston, Houston, TX, USA; 2 Department of Biostatistical Sciences, Wake Forest University School of Medicine, Winston-Salem, NC, USA; 3 Department of Pediatrics, Wake Forest University School of Medicine, Winston-Salem, NC, USA; 4 Department of Social Sciences and Health Policy, Wake Forest University School of Medicine, Winston-Salem, NC, USA; The Catholic University of Korea, Korea

**Keywords:** Accreditation, Graduate medical education, Program evaluation, Workforce, United States

## Abstract

**Purpose:**

Declining fill rates in US pediatric residency and subspecialty programs make trainee retention especially important. Attrition, defined as transfers, withdrawals, dismissals, unsuccessful completions, or deaths, disrupts program function and impacts the pediatric workforce pathway. This study aimed to evaluate attrition trends among pediatric residents and fellows in Accreditation Council for Graduate Medical Education (ACGME)-accredited programs from 2007 to 2020.

**Methods:**

This repeated cross-sectional study analyzed publicly available ACGME Data Resource Book records. Attrition rates and 95% confidence intervals (CIs) were calculated overall and by subspecialty. Logistic regression assessed temporal changes; odds ratios (ORs) compared 2020 to 2007.

**Results:**

From 2007–2020, the number of pediatric residents increased from 8,145 to 9,419 and fellows from 2,875 to 4,279. Aggregate annual resident attrition averaged 1.71% (range, 0.93%–2.64%), and fellow attrition ranged from 12.39%–30.87%. Transfer rates declined from 18.05 to 5.20 per 1,000 trainees (P<0.0001), withdrawals from 5.65 to 2.76 (P=0.030), and dismissals from 3.14 in 2010 to 1.27 in 2020 (P=0.0068). Odds of unsuccessful completion significantly decreased in categorical pediatrics (OR, 0.41; 95% CI, 0.29–0.58), pediatric cardiology (OR, 0.08; 95% CI, 0.01–0.64), pediatric critical care (OR, 0.14; 95% CI, 0.06–0.35), and neonatal-perinatal medicine (OR, 0.46; 95% CI, 0.20–1.08).

**Conclusion:**

Although attrition has improved, premature trainee loss can still disrupt program operations and threaten workforce development. Attrition may reflect educational environment quality, support structures, or selection processes. Greater data transparency is needed to understand demographic trends and inform equitable retention strategies, ultimately strengthening training programs and sustaining the United States pediatric workforce.

## Graphical abstract

[Fig f2-jeehp-22-24]

## Introduction

### Background

Applications to pediatric residency and fellowship programs have steadily declined over the past 6 years [1]. In 2023, approximately 55% of US medical school graduates who submitted applications for categorical pediatric residency positions also applied to another specialty (“cross-specialty applications”) [2]. In the 2024 Match, pediatric residencies increased the number of offered positions by 93 compared to the previous year. Of the 3,139 positions offered, only 92% were filled—down from 97.1% the prior year—leaving 252 positions unfilled, 164 more than the year before. The percentage of US MD (Doctor of Medicine) seniors matching into categorical pediatrics declined to 47.6%, a 7.2% decrease from 2023 [3]. Pediatric subspecialty fellowships reflected similar trends, with only 79% of offered positions filled in 2024—a 5.5% decrease compared to the previous year [1].

Although recent efforts have aimed to enhance recruitment into pediatrics [4-7], retention of trainees is equally critical to sustaining the pediatric workforce. Training programs must not only attract but also retain trainees, as the loss of a resident or fellow can negatively impact educational continuity, clinical coverage, team morale, duty hour compliance, and program funding [8]. Attrition may reflect the quality of the educational environment, the effectiveness of support structures, or the robustness of selection processes. Therefore, understanding trends and reasons for attrition can guide program evaluation and reform.

From a systems perspective, trainee attrition can serve as a key indicator of training program effectiveness. Tracking attrition offers valuable insights for educational quality assurance, curriculum improvement, and policy development at both the institutional and national levels. Despite its importance, little research has examined attrition among pediatric residents and fellows.

### Objectives

The primary objective of this study was to evaluate pediatric resident and fellow attrition trends from 2007 to 2020.

## Methods

### Ethics statement

The study was approved by the Institutional Review Board (IRB) of Wake Forest University School of Medicine (IRB00101797).

### Study design

This repeated cross-sectional study of pediatric resident and fellow attrition utilized publicly available data from the Accreditation Council for Graduate Medical Education (ACGME) Data Resource Books from 2007 to 2020, the most recent year available at the time of analysis [9].

### Setting

Data search was done from September 2023 to October 2023.

### Participants

We excluded residents from combined residencies not restricted to pediatric trainees and subspecialties in which completion of a pediatric residency was not required, such as the pediatric surgical subspecialties. Attrition was defined by the ACGME as a pediatric trainee (categorical residents and subspecialty fellows) who left their training program during a specific year.

### Variables

Attrition reasons included transfer to another program, withdrawal, dismissal, unsuccessful completion, or death. The transfer data did not indicate the type of program to which the trainee transferred. Unsuccessful completion and death were not tracked until the year 2009. No demographic data were available for the dataset.

### Data sources/measurement

Data source was the ACGME Data Resource Books from 2007 to 2020. The aggregate rate of resident overall attrition, transfer rate of all pediatric trainees, withdrawal rate of all pediatric trainees, and overall rate of unsuccessful completion were measured.

### Bias

Since all target subjects were included in the analysis, there was no selection bias.

### Statistical methods

The rate of attrition and exact 95% confidence intervals (CIs) were calculated for the overall group and by specialty. To assess change over time, logistic regression was used to model the rates. If overall significance was observed, odds ratios (ORs) and 95% CIs comparing 2020 to 2007 were presented. Results were considered statistically significant at a 2-tailed P-value <0.05. SAS ver. 9.4 (SAS Institute Inc.) was used for all analyses.

## Results

### Participants

Status of all target residents from 2007 to 2020 were listed in Dataset 1.

### Main results

The total number of pediatric residents in ACGME-accredited programs increased from 8,145 to 9,419 over the period spanning 2007 to 2020. The number of pediatric fellows in ACGME accredited programs increased from 2,875 to 4,279 over this same period.

The aggregate rate of resident overall attrition was 1.71% per year ranged from the lowest in 2020 at 0.93%, the highest at 2.64% in 2009. The aggregate rate of pediatric fellow attrition per year ranged from the lowest in 2020 at 12.39, the highest at 30.87 in 2008.

**Fig. 1.**
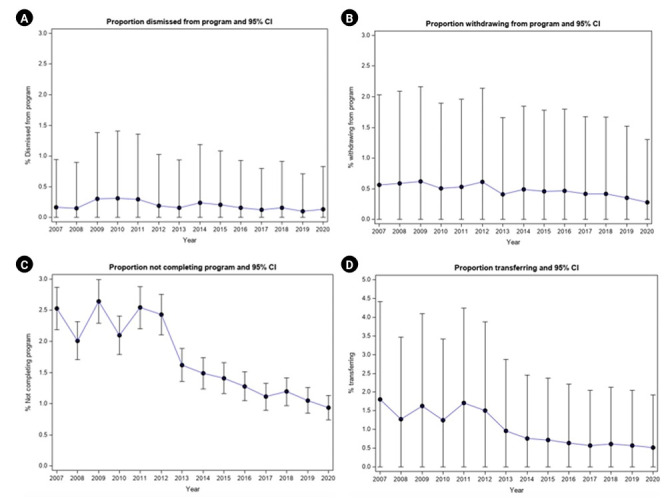
Proportion of United States pediatric trainees dismissed; withdrawing; not completing; and transferring. (A) Proportion dismissed from program and 95% confidence interval (CI). (B) Proportion withdrawing from program and 95% CI. (C) Proportion not completing program and 95% CI. (D) Proportion transferring and 95% CI.

The overall transfer rate of all pediatric trainees was 10.14/1,000 (18.05 in 2007 to 5.20 in 2020; P<0.0001). The overall withdrawal rate of all pediatric trainees was 4.74/1,000 (5.65 in 2007 to 2.76 in 2020; P=0.030) and the overall dismissal rate was 1.87/1,000 (1.60 in 2007, 1.27 in 2020, peaking at 3.14 in 2010; P=0.0068) (Table 1, Fig. 1).

The overall rate of unsuccessful completion of all pediatric trainees was 0.30/1,000 (0.97 in 2009, peaking to 1.04 in 2012, and dropping to 0.11 in 2014; P<0.0001). Deaths were rare and not different over the years with a rate of 0.12/1,000 (the highest rate was 0.35 in 2012, and low of 0 in several years: 2009, 2011, 2014, 2016, and 2017; P=0.99).

Additionally, the odds of unsuccessful completions significantly decreased during the study period for categorical pediatric residency training (P<0.0001 overall, comparing 2020 to 2007; OR, 0.41; 95% CI, 0.29–0.58), pediatric cardiology (P=0.0086 overall, 2020 vs. 2007; OR, 0.08; 95% CI, 0.01–0.64), pediatric critical care (P<0.0001; OR, 0.14; 95% CI, 0.06–0.35), and neonatal-perinatal medicine (P=0.012 overall, 2020 vs. 2007; OR, 0.46; 95% CI, 0.20–1.08) fellowships.

## Discussion

### Key results

From 2007–2020, ACGME pediatric residents increased from 8,145 to 9,419 and fellows from 2,875 to 4,279. Resident attrition averaged 1.71% annually. Transfer, withdrawal, and dismissal rates declined. Unsuccessful completion odds fell significantly across residency and fellowships, with notable reductions in pediatric cardiology, critical care, and neonatal-perinatal programs during the period.

### Interpretation/comparison with previous studies

Our study demonstrated that pediatric trainee overall attrition rates decreased over time between 2007 and 2020. The most common reason for attrition was “transfer to another program.” Additionally, the odds of unsuccessful completions significantly declined in pediatric cardiology, pediatric critical care, and neonatal-perinatal medicine fellowships. These findings provide timely insight into workforce sustainability, especially given recent concerns about declining application and fill rates in both pediatric residencies and fellowships [10].

While it is unclear whether transferred trainees remained in pediatrics or switched specialties, prior research in obstetrics-gynecology suggests that approximately one-third of such transfers result in specialty change [11]. More concerning are cases of dismissal, which likely reflect serious disruptions in training. Unpublished data suggest that residents underrepresented in medicine (URiM) are disproportionately represented among dismissals [12], underscoring the need to examine systemic contributors to inequitable retention.

The decrease in overall attrition over time, especially following 2011, may reflect the impact of ACGME duty hour restrictions implemented that year. Similar temporal trends have been observed in neurosurgery and other specialties [13,14]. The reduction in overall attrition within high-demand, high-reward subspecialties such as cardiology and critical care may also reflect improved incentives for completion, including competitive compensation and high fill rates [15].

### Limitations

Interpretation of these findings is limited by the lack of granular data. Publicly available ACGME datasets do not currently include variables such as race, ethnicity, gender identity, citizenship status, or year of training at the time of attrition. Nor do they distinguish between intra-specialty and inter-specialty transfers. Limitations exist in using aggregate data for subspecialty-level analysis given the smaller sample sizes. Additionally, there is no available qualitative data, such as trainee exit interviews. These data gaps hinder our ability to assess equity and to tailor retention strategies. Causality cannot be inferred and reasons for attrition cannot be determined from the dataset.

### Educational implications/suggestions

Attrition trends offer a valuable lens for evaluating the effectiveness of residency and fellowship programs. Program directors, Designated Institutional Officials, and policymakers could use attrition data as part of ongoing quality assurance efforts and institutional benchmarking. For example, identifying high-risk timepoints (e.g., transitions between training years) or subgroups (e.g., URiM trainees) could help institutions implement proactive support structures. These structures might include mentorship programs, confidential systems for raising concerns, structured wellness initiatives, recognition of positive contributions, and support for personal needs such as psychological services, paid leave, and time for medical appointments. Training programs should also evaluate their Clinical Competency Committees and assessment practices to mitigate bias, and invest in faculty development focused on microaggressions, implicit bias, and discrimination. Institutions can further enhance retention by ensuring appropriate clinical staffing, reducing administrative burden through technology, and advocating for policies that regulate duty hours, support flexible scheduling, offer competitive compensation, and provide loan repayment during training. Transparency around ACGME attrition data, including demographic breakdowns and reasons for departure, would enable more precise, equity-informed strategies for workforce retention. Advocacy for improved data access is essential for developing policy interventions and targeted educational reforms that reduce preventable loss from the physician pathway.

### Conclusion

Pediatric trainee attrition rates have declined over time, suggesting progress in supporting trainee retention. Nevertheless, any premature loss remains disruptive—impacting individual career trajectories, program function, and the broader pediatric workforce pipeline. Attrition should be viewed not only as a personnel issue but also as an indicator of training program effectiveness. Improved data transparency, particularly around demographic and program-level factors, is essential to identify systemic barriers, guide targeted interventions, and promote equity in trainee retention. Future efforts should prioritize both reducing preventable attrition and using attrition data to inform educational improvement and workforce planning.

## Figures and Tables

**Figure f2-jeehp-22-24:**
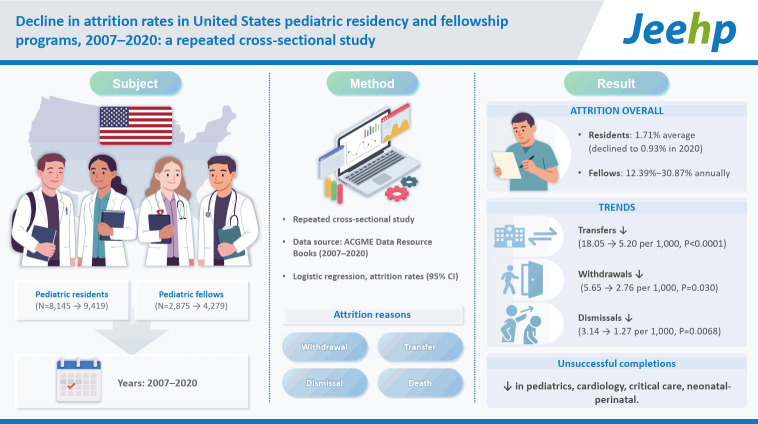


**Table 1.** United States pediatric graduate medical education trainee attrition rates from 2007 to 2020

	Total trainees	Trainee attrition in 2007	% Rate	95% CI	Total trainees	Trainee attrition in 2020	% Rate	95% CI	P-value
Adolescent medicine	59	1	1.69	(0–9.09)	97	1	1.03	(0–5.61)	0.88
Child abuse pediatrics	NA	NA			44	1	2.27	(0.1–12.0)	NA
Clinical informatics	NA	NA			19	0	0	(0–17.6)	NA
Developmental-behavioral pediatrics	69	1	1.45	(0–7.81)	114	2	1.75	(0.21–6.19)	0.86
Neonatal-perinatal medicine	571	14	2.45	(1.35–4.08)	783	9	1.15	(0.53–2.17)	0.012
Pediatric cardiology	304	8	2.63	(1.14–5.12)	468	1	0.21	(0–1.18)	0.0086
Pediatric critical care medicine	348	24	6.9	(4.47–10.06)	575	6	1.04	(0.38–2.26)	<0.0001
Pediatric emergency medicine	266	4	1.5	(0–3.81)	434	2	0.46	(0.06–1.65)	0.37
Pediatric endocrinology	216	6	2.78	(1.03–5.95)	230	8	3.48	(1.51–6.74)	0.62
Pediatric gastroenterology	216	3	1.39	(0–4.01)	329	1	0.30	(0–1.68)	0.092
Pediatric hematology/oncology	354	7	1.98	(0.80–4.03)	475	6	1.26	(0.46–2.73)	0.3
Pediatric hospital medicine	NA	NA			115	3	2.61	(0.54–7.43)	NA
Pediatric infectious diseases	163	5	3.07	(1.00–7.01)	175	8	4.57	(1.99–8.81)	0.88
Pediatric nephrology	103	8	7.77	(3.41–14.73)	120	2	1.67	(0.20–5.89)	0.12
Pediatric pulmonology	130	3	2.31	(0.48–6.60)	176	2	1.14	(0.14–4.04)	0.52
Pediatric rheumatology	66	2	3.03	(0.37–10.52)	88	1	1.14	(0–6.17)	0.67
Pediatric transplant hepatology	NA	NA			11	0	0	(0–28.5)	NA
Sports medicine	10	0	0	(0–30.85)	26	0	0	(0–13.2)	>0.99
Pediatrics	8,145	206	2.53	(2.20–2.89)	9,419	88	0.93	(0.75–1.15)	<0.0001

CI, confidence interval; NA, not applicable.

## Footnotes

**Data availability**
Data files are available from https://doi.org/10.7910/DVN/MYN29A Dataset 1. The data file contains the raw data.

## Supplementary materials

Supplement 1. Audio recording of the abstract.
